# Optimization of Biomass Accumulation and Production of Phenolic Compounds in Callus Cultures of *Rhodiola rosea* L. Using Design of Experiments

**DOI:** 10.3390/plants11010124

**Published:** 2022-01-02

**Authors:** Anna A. Erst, Anastasia A. Petruk, Andrey S. Erst, Denis A. Krivenko, Nadezhda V. Filinova, Svetlana Y. Maltseva, Maxim S. Kulikovskiy, Evgeny V. Banaev

**Affiliations:** 1 Central Siberian Botanical Garden, Siberian Branch of Russian Academy of Sciences, 630090 Novosibirsk, Russia; pet.a@mail.ru (A.A.P.); erst_andrew@yahoo.com (A.S.E.); alnus2005@mail.ru (E.V.B.); 2 Laboratory of Plants Systematics and Phylogeny, National Research Tomsk State University, 634050 Tomsk, Russia; 3 Siberian Institute of Plant Physiology & Biochemistry, Siberian Branch of Russian Academy of Sciences, 664033 Irkutsk, Russia; krivenko.irk@gmail.com (D.A.K.); filinova_nv@mail.ru (N.V.F.); 4 K.A. Timiryazev Institute of Plant Physiology, Russian Academy of Sciences, 127276 Moscow, Russia; svetadm32@gmail.com (S.Y.M.); max-kulikovsky@yandex.ru (M.S.K.)

**Keywords:** roseroot, in vitro culture, design of experiments, nitrogen source, plant growth regulator, methyl jasmonate, HPLC, phenolic compound, histochemistry

## Abstract

*Rhodiola rosea* L. is a valuable medicinal plant with adaptogenic, neuroprotective, antitumor, cardioprotective, and antidepressant effects. In this study, design of experiments methodology was employed to analyze and optimize the interacting effects of mineral compounds (concentration of NO_3_^−^ and the ratio of NH_4_^+^ to K^+^) and two plant growth regulators [total 6-benzylaminopurine (BAP) and α-naphthylacetic acid (NAA) concentration and the ratio of BAP to NAA] on the growth and the production of total phenolic compounds (TPCs) in *R. rosea* calluses. The overall effect of the model was highly significant (*p* < 0.0001), indicating that NH_4_^+^, K^+^, NO_3_^−^, BAP, and NAA significantly affected growth. The best callus growth (703%) and the highest production of TPCs (75.17 mg/g) were achieved at an NH_4_^+^/K^+^ ratio of 0.33 and BAP/NAA of 0.33, provided that the concentration of plant growth regulators was 30 μM and that of NO_3_^−^ was ≤40 mM. According to high-performance liquid chromatography analyses of aerial parts (leaves and stems), in vitro seedlings and callus cultures of *R. rosea* contain no detectable rosarin, rosavin, rosin, and cinnamyl alcohol. This is the first report on the creation of an experiment for the significant improvement of biomass accumulation and TPC production in callus cultures of *R. rosea*.

## 1. Introduction

The application of plant in vitro systems as a sustainable platform for the biotechnological production of pharmaceuticals is a promising alternative to the traditional pipeline. In vitro systems possess numerous advantages, including biosynthesis of safe metabolites according to good manufacturing practices (GMP) and independence from environmental factors [1,2,3]. In addition, this approach does not threaten natural populations of rare and endangered plant species. One of the species currently in demand for the biotechnological production of natural substances, having adaptogenic properties and various medicinal effects [4,5,6], is *Rhodiola rosea* L. from the Crassulacea family. Recently, *R. rosea* was actively used in the manufacture of various dietary supplements [7]; large volumes of harvesting in the wild and a slow rate of renewal have put this species on the brink of extinction. Notably, most of the products on the market are based on the raw material collected from wild populations in the Altai region (Russia) [5]. It is fairly well documented that the standard in vitro culture of *R. rosea* is not efficient enough to compete with wild plants with respect to the accumulation of active ingredients. In almost all earlier reports, researchers admit the lack or only traces of the important compounds in their in vitro experiments with roseroot [8,9,10,11]. In in vitro cultures, a significant enhancement of the production of rosin and its derivatives is observed when the cultures are fed with a precursor: cinnamyl alcohol [2,8,12]. In *R. rosea* compact callus aggregate cultures, the observed rosin and rosarin content is even higher than that in field-cultivated plants, while the rosavin level is five times lower. Recently developed hairy root cultures of *Rhodiola kirilowii* (Regel) Maxim. supplemented with cinnamyl alcohol exhibit a higher potential for the production of rosin and its derivatives in comparison to field-cultivated plants [13]. It has been shown that light quality has a stimulatory effect on secondary-metabolite production in callus cultures of *R. imbricate* Edgew [14]. Therefore, new approaches should be developed to overcome the shortage of active ingredients in in vitro cultures.

In this study, the design of experiments (DoE) methodology was utilized to examine the parameters that affect callus biomass accumulation and production of phenolic compounds by *R. rosea*. DoE is a statistical methodology that allows simultaneous testing of multiple factors to understand and improve complex systems [15,16].

Mineral nutrients are some of the most basic components of plant tissue culture media. Nitrogen (N) in the form of NH_4_^+^ or NO_3_^−^ is the dominant mineral nutrient in most tissue culture formulations [17]. The culture of isolated plant tissues is autotrophic with respect to the N source. From inorganic N sources, tissues synthesize all organic nitrogenous compounds necessary for normal physiological processes [18]. The concentration and form of N in tissue culture media have a significant influence on cell growth and differentiation [19]. The most common N forms used in tissue culture growth media are NO_3_^−^ and NH_4_^+^. The effects of N may be dependent on either the total N concentration or the proportion of NO_3_^−^ and NH_4_^+^. In general, NO_3_^−^ is the favored form for N assimilation in most plants; NH_4_^+^ is sometimes not required and at high concentrations may be toxic [19,20]. For most plants, a combination of NO_3_^−^ and NH_4_^+^ is better than either NO_3_^−^ or NH_4_^+^ as a sole source of N. Changes of NH_4_NO_3_ and KNO_3_ concentrations will alter the concentrations and proportions of K^+^, NO_3_^−^, and NH_4_^+^ in the culture medium [21]. In plant tissue culture media, N is present as various salts and varying the proportions of salts creates the problem of ion confounding between the effect of the N source and the effect of the counter ion in that salt. Varying the NH_4_^+^/K^+^ ratio and the total nitrate ion concentration NO_3_^−^ in a two-component mixture facilitates the direct estimation of mineral nutrients’ effects without the ion confounding of a salt-based approach [17]. Computer-aided experimental design helps formulate practical treatments consisting of several factors or mixtures for studying the effects of complicated systems, in contrast to the traditional studies or factorial designs [22].

Various concentrations and combinations of plant growth regulators have been used to obtain callus and suspension cultures of *Rhodiola* species. For the induction of callus formation in these species, a combination of cytokinins and auxins is required [10,23,24,25,26,27,28,29,30,31]. 6-benzylaminopurine (BAP) is one of the most widely used plant growth regulators for in vitro culture of *Rhodiola*. Cotyledons, hypocotyl, leaves, apical buds, and internode fragments, inoculated on media containing BAP (0.2–3 mg/L) in combination with indole-3-acetic acid (IAA; 0.1 mg/L) [24], α-naphthylacetic acid (NAA; 0.5–3 mg/L) [10,28], or 2,4-dichlorophenoxyacetic acid (2,4-D; 0.5–3.0 mg/L) [25,31] have been the most appropriate explant types and media constituents for induction of well-growing calluses [5].

Numerous authors have demonstrated that the addition of an elicitor (biotic or abiotic) to a culture medium significantly increases the production of secondary metabolites in vitro by triggering a metabolic cascade [32,33]. Jasmonic acid and methyl jasmonate (MJ) are recognized as effective elicitors that trigger the biosynthesis of secondary defense compounds in callus and suspension cultures by activating the genes of secondary metabolism [34]. For example, jasmonic acid has been shown to enhance phenolic-compound production and bioactivity in a suspension culture of *R. imbricate* [28].

In the present study, the DoE approach was used to explore the relations among multiple factors in a new rapid culture method and their influence on process outcomes. DoE is particularly useful for examining interactions among factors that cannot be predicted by experiments designed to test one factor at a time (OFAT) [35]. In our study, four factors were tested that influence biomass accumulation and the production of phenolic compounds in the callus culture of *R. rosea*: ratio NH_4_^+^/K^+^, NO_3_^−^ concentration, ratio BAP/NAA, and BAP + NAA concentration. In addition, in this study, we evaluated the impact of MJ in callus culture under optimal nutrient conditions.

## 2. Results

A summary of ANOVA (analysis of variance), the lack-of-fit test, and three R^2^ statistics for % fresh weight increase and dry weight is presented in Table 1. The percent fresh weight increase in the biomass of *R. rosea* callus after 45 days of cultivation varied from 14% to 703%. The result of the lack-of-fit test was not significant (*p* = 0.8180), indicating that additional variation in the residuals could not be removed with a better model. R^2^, adjusted R^2^, and predicted R^2^ ranged from 0.92 to 0.98. The effect of the overall model was highly significant (*p* < 0.0001), indicating that NH_4_^+^, K^+^, NO_3_^−^, BAP, and NAA significantly affected the growth. ANOVA revealed 17 significant terms, 12 of which had *p*-values < 0.0001. Dry weight accumulation ranged from 0.05 to 0.26 g. R^2^, adjusted R^2^, and predicted R^2^ ranged from 0.73 to 0.91, indicating good agreement among these five values. The overall effect of the model was highly significant (*p* < 0.0001), indicating significant factor effects on dry weight by these three ions and plant growth regulators. ANOVA revealed nine significant terms, three of which had *p*-values < 0.0001 (Table 1).

### 2.1. Effects of the NH_4_^+^/K^+^ Ratio and NO_3_^−^ Concentration on Callus Culture

**Figure 1:**
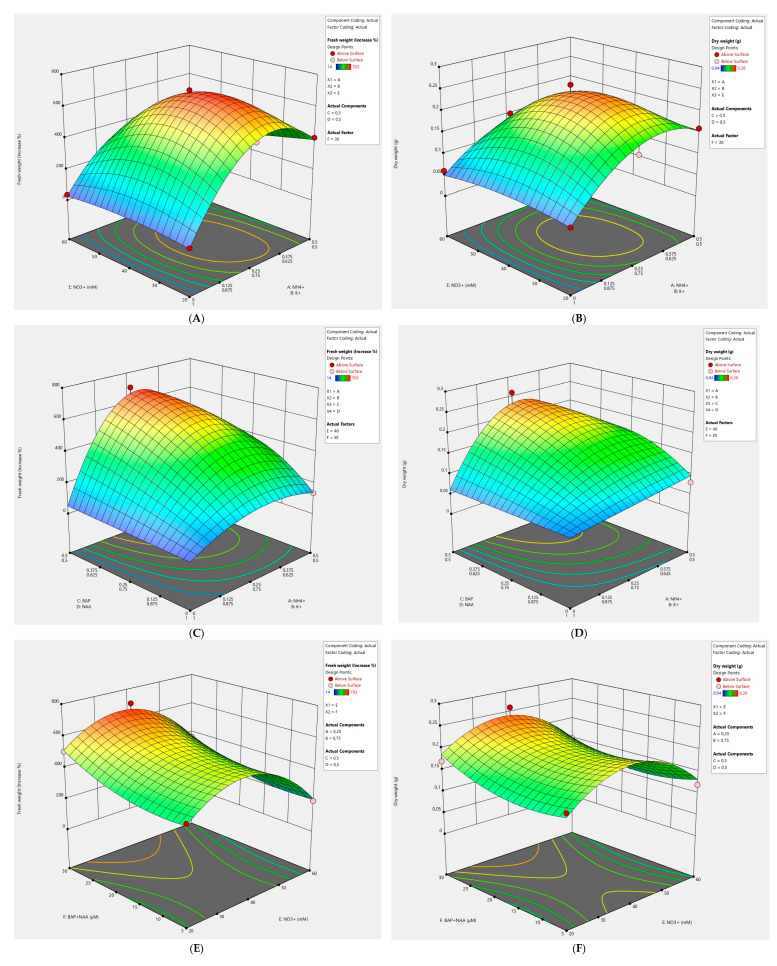
Three-dimensional (3D) response surface plots showing the effects of various factors on *R. rosea* callus fresh and dry weight. (**A**,**B**) The impact of the NH_4_^+^/K^+^ ratio and NO_3_^−^. (**C**,**D**) The influence of the BAP/NAA ratio and NH_4_^+^/K^+^. (**E**,**F**) The effect of the concentrations of BAP + NAA and NO_3_^−^.

Callus growth in the control treatment group, with the NH_4_^+^/K^+^ ratio at 1.0 and 40 mM NO_3_^−^, was moderate (approximately average; Figure 1A,B). The highest values were obtained with the NH_4_^+^/K^+^ ratio of 0.33 and 20–40 mM NO_3_^−^. The influence of the Linear x Linear Mixture component was highly significant for fresh and dry weight. The Linear x Linear Mixture component compares the responses at the extreme ends (vertices) of the mixture design space. This means that growth at the points corresponding to the NH_4_^+^/K^+^ ratio of 0/1 was compared to growth at the points corresponding to the NH_4_^+^/K^+^ ratio of 0.5/0.5. We noted that good growth of callus culture requires NH_4_^+^ > 0. The constructed model showed that the concentration of NO_3_^−^ has a smaller effect on the growth of fresh and dry biomass in comparison with the NH_4_^+^/K^+^ ratio.

### 2.2. Effects of Ratios BAP/NAA and NH_4_^+^/K^+^ on Callus Culture

The impact of the interaction between ratios BAP/NAA and NH_4_^+^/K^+^ was very highly significant toward the % fresh weight increase and dry weight of callus culture of *R. rosea*. The largest values were achieved at the NH_4_^+^/K^+^ ratio of 0.33 and BAP/NAA 0.33–1.00, provided that the concentration of plant growth regulators was 30 μM and the content of NO_3_^−^ was not more than 40 mM (Figure 1C,D). A change in the parameters of the model showed that with a decrease in the content of NO_3_^−^, there is a shift of the maximum toward the NH_4_^+^/K^+^ ratio of 1.0. Good growth of callus culture required BAP > 0.

### 2.3. Effects of BAP + NAA and NO_3_^−^ Concentrations on Callus Culture

The best growth was achieved at high concentrations of plant growth regulators (30 µM) and NO_3_^−^ concentrations of 20–40 mM (Figure 1E,F). We also noted a large increase in dry biomass at a low concentration of sum BAP (5 μM) and NAA (5 μM). The constructed model indicated that peak values were not reached in the experiment.

### 2.4. Histochemical Analysis

**Figure 2:**
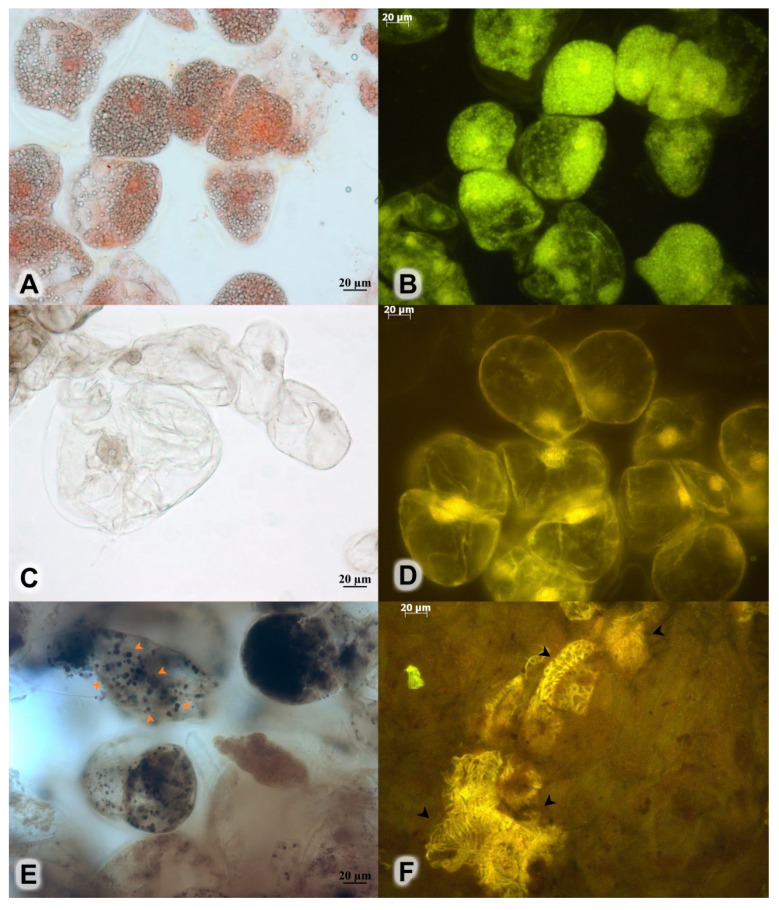
Callus culture of *R. rosea* cultivated on a modified MS medium at the NH_4_^+^/K^+^ ratio of 0.33, 40 mM NO_3_^−^, the BAP/NAA ratio of 1.0, and concentrations of plant growth regulators at 30 μM. (**A**) Numerous small vacuoles in immature cells seen under a light microscope and (**B**) under a fluorescence microscope. (**C**) Mature cells with a single vacuole under the light microscope and (**D**) under the fluorescence microscope. (**E**) Lugol’s solution for starch (orange arrows). (**F**) Fragments of spiral vessels as seen under the fluorescence microscope (black arrows).

The callus culture obtained under optimal culture conditions was semi-friable yellowish green. Many of the large calluses contained cavities at their centers. Histological analysis revealed that the calluses consisted of actively dividing cells with two types of vacuoles: numerous small vacuoles or a single central one (Figure 2A–D). Small vacuoles are characteristic of immature cells and large vacuoles of mature cells. Lugol’s staining showed some accumulation patterns of starch granules in the callus culture (Figure 2E). Starch granules were previously found by us only in the cells of the root and rhizome, while leaves and stems had no starch-containing cells [36]. Cells of the 45-day-old callus did not yield positive signals in a ferric chloride reaction for phenolic compounds. We previously demonstrated that roots and rhizomes tested positive for phenolic compounds with this reagent [36]. In the present study, we found chaotic xylem elements in compacted parts of the callus cultures (Figure 2F).

### 2.5. Biochemical Analysis

#### 2.5.1. The Profile and Levels of Phenolic Compounds in Callus Culture and Seedlings (Control 1)

**Figure 3:**
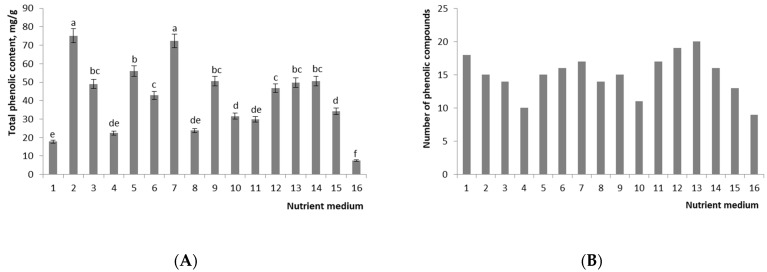
Effect of composition of the nutrient medium and different concentrations of MJ on (**A**) total phenolic content and (**B**) number of phenolic compounds in callus cultures of *R. rosea.* Values are mean ± standard error (vertical error bars) of three replicates. Means with similar letters are not significantly different at *p* ≤ 0.05 according to LSD test. Legend. Treatment group 1: NH_4_^+^/K^+^ 0.33, NO_3_^−^ 20 mM, BAP/NAA 1.0, BAP + NAA 5 μM; 2: NH_4_^+^/K^+^ 0.33, NO_3_^−^ 20 mM, BAP/NAA 0.33, BAP + NAA 30 μM; 3: NH_4_^+^/K^+^ 0.33, NO_3_^−^ 20 mM, BAP/NAA 1.0, BAP + NAA 30 μM; 4: NH_4_^+^/K^+^ 0.33, NO_3_^−^ 40 mM, BAP/NAA 0.33, BAP + NAA 17.5 μM; 5: NH_4_^+^/K^+^ 0.33, NO_3_^−^ 40 mM, BAP/NAA 0.33, BAP + NAA 30 μM; 6: NH_4_^+^/K^+^ 0.33, NO_3_^−^ 40 mM, BAP/NAA 1.0, BAP + NAA 30 μM; 7: NH_4_^+^/K^+^ 0.33, NO_3_^−^ 60 mM, BAP/NAA 0.33, BAP + NAA 30 μM; 8: NH_4_^+^/K^+^ 0.33, NO_3_^−^ 60 mM, BAP/NAA 1.0, BAP + NAA 30 μM; 9: NH_4_^+^/K^+^ 1.0, NO_3_^−^ 20 mM, BAP/NAA 0.33, BAP + NAA 17.5 μM; 10: NH_4_^+^/K^+^ 1.0, NO_3_^−^ 20 mM, BAP/NAA 0.33, BAP + NAA 30 μM; 11: NH_4_^+^/K^+^ 1.0, NO_3_^−^ 20 mM, BAP/NAA 1.0, BAP + NAA 30 μM; 12: NH_4_^+^/K^+^ 1.0, NO_3_^−^ 30 mM, BAP/NAA 1.0, BAP + NAA 17.5 μM; 13: NH_4_^+^/K^+^ 1.0, NO_3_^−^ 40 mM, BAP/NAA 1.0, BAP + NAA 30 μM; 14: NH_4_^+^/K^+^ 0.33, NO_3_^−^ 40 mM, BAP/NAA 1.0, BAP + NAA 30 μM, MJ 100 μM; 15: NH_4_^+^/K^+^ 0.33, NO_3_^−^ 40 mM, BAP/NAA 1.0, BAP + NAA 30 μM, MJ 200 μM; 16: control 1.

Biochemical analysis was carried out only for well-growing callus cultures (an increase in fresh biomass of more than 300%) and for callus grown on the standard MS medium. High-performance liquid chromatography (HPLC; suitable for isolating a complex set of phenolic compounds) detected 27 phenolic compounds in the callus culture and nine phenolic compounds in seedlings of *R. rosea* (control 1) (Table S1). Total phenolic compound (TPC) concentration varied among callus cultures from 14.9 to 71.6 mg/g. The number of phenolic compounds varied from 10 to 20; in control 1, these values were 6.5 mg/g and 9, respectively (Figure 3).

The highest content of TPC was observed in calluses grown on media with the NH_4_^+^/K^+^ ratio of 0.33 and BAP/NAA of 0.33 and a concentration of plant growth regulators of 30 μM (56.01–75.17 mg/g), which is 1.1–1.5 times higher than that in the standard MS medium supplemented with 15 μM BAP and 15 μM NAA (49.73 mg/g) (Figure 3 and Figure S1). The lowest amount of phenolic compounds was found in control 1 and in the sample cultured on the medium with the NH_4_^+^/K^+^ ratio of 0.33, BAP/NAA of 1.0, with the lowest concentration of plant growth regulators, i.e., 5 μM. The level of biosynthesis of TPCs on the nutrient medium with the highest increase in callus biomass was 13% lower than that on the standard MS medium (42.85 and 49.73 mg/g, respectively) (Figure 3).

The treatment of callus cultures with an elicitor led to qualitative and quantitative changes in the profile of phenolic compounds (Figure 3 and Figure S2). MJ added at 100 μM gave a 119% increase relative to nonelicited cultures. We demonstrated that when callus cultures were exposed to an elicitor, four phenolic compounds emerged that were absent in nonelicited cultures; retention times were 13.6, 30.0, 33.3 and 46.0 (Table S1).

**Figure 4:**
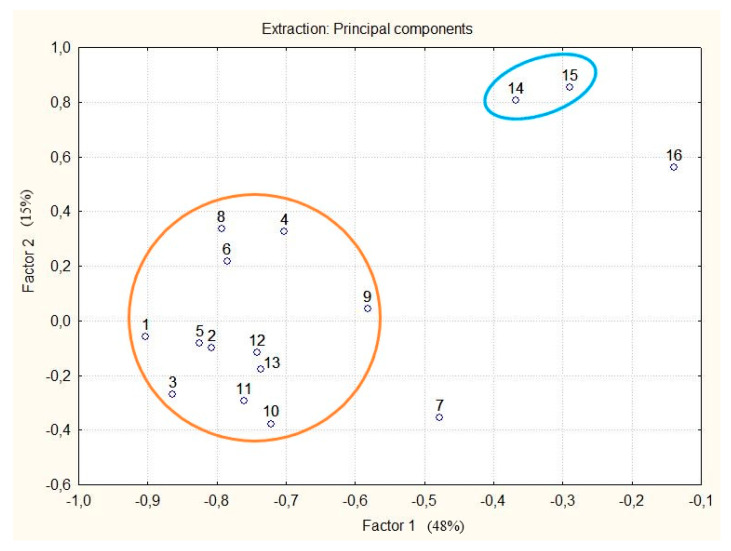
The principal component analysis (PCA) plot showing two clusters of callus cultures and control 1 of *R. rosea*. Blue ellipse: callus culture after treatment with MJ; orange ellipse: all other callus cultures in the experiment, except No. 7; No. 7: callus culture obtained on the medium that had NH_4_^+^/K^+^ of 0.33, NO_3_^−^ at 60 mM, BAP/NAA 0.33, BAP + NAA 30 μM; No. 16: control 1. Note: see Figure 3.

Multivariate statistical analysis was performed to classify the differences in phenolic compounds among callus cultures. Unsupervised principal component analysis (PCA) revealed two major clusters (Figure 4). Two principal components, PC1 and PC2, together accounted for 63% of the total variance. Control sample 1, sample No. 7 and samples treated with MJ can be clearly distinguished from the main cluster. Sample No. 7 is characterized by richer qualitative and quantitative composition (TPCs at 72.27 mg/g, 17 phenolic compounds).

#### 2.5.2. The Profile and Levels of Phenolic Compounds in Immature Plants (Control 2)

Five-year-old cultivated plants of *R. rosea* served as control 2 in the experiment when the biochemical composition was analyzed. HPLC (suitable for isolating a complex set of phenolic compounds) detected 10 phenolic compounds in the rhizomes and roots and 21 phenolic compounds in the above-ground part of *R. rosea* (Table 2). Compounds No. 5–8, identified as cinnamyl alcohol and its derivatives (phenylpropanoids), were found only in underground organs. Their observed maxima are typical for rhizomes: rosarin 16.33 mg/g, rosavin 41.73, rosin 25.10, and cinnamyl alcohol 42.74 mg/g (Table 2, Figure S3). The total content of phenolic compounds was 159.09 mg/g in the rhizomes and 88.68 mg/g in the roots.

Our study revealed that none of the callus culture samples, including the MJ-treated one, control 1, and above-ground parts of *R. rosea* contained rosin, rosavin, rosarin, and cinnamyl alcohol.

## 3. Discussion

Using DoE, correct nutrient combinations were identified, along with the effects of their interactions with the other environmental parameters: plant growth regulators. Similar methods have recently been used for optimizing callus cultures of various plant species [35,37,38]. This method has two advantages over the more common factorial design: DoE allows us to investigate the effects of independent variables between the actual experimental data points and allows a researcher to easily increase the number of experimental variables to more than five, which is not practical in typical factorial designs. Our findings may facilitate the application of DoE to tissue culture optimization and the in vitro production of secondary metabolites, where an understanding of the complex interactions among plant growth regulators and growth medium nutrients is sought. Nitrogen quantity and form have been the subject of many growth medium optimization studies [39,40,41,42,43]. The optimum nitrate concentration is reported to be 20–30 mM for both growth and taxol production in cell cultures of *Taxus yunnanensis* Cheng et al. [40]. In an experiment on the NH_4_^+^/NO_3_^−^ ratio in the culture medium, ginsenoside production in the adventitious roots of *Panax ginseng* C.A. Meyer was affected by NH_4_^+^/ NO_3_^−^ ratios in the culture medium, showing the greatest productivity at 18.5 mM NO_3_^−^ without NH_4_^+^ [39]. Biomass growth and azadirachtin production of *Azadirachta indica* (A. Juss) suspension cultures are significantly improved in a medium with a high NH_4_^+^/NO_3_^−^ ratio [41]. According to our data, the NH_4_^+^/K^+^ ratio is a crucial factor for biomass accumulation and production of TPCs in the callus culture of *R. rosea*. The optimal response was seen at the NH_4_^+^/K^+^ ratio of 0.33 and 20–40 mM NO_3_^−^.

Plant growth regulators are one of the most important factors owing to their important regulating role in plant physiology and biochemistry [44]. An appropriate proportioning of cytokinins and auxins can maintain the balance between differentiation and dedifferentiation and achieve the goal of rapid proliferation for plant cells in vitro [45]. To obtain callus cultures of *Rhodiola* species, cytokinin BAP is most often employed in combination with various auxins. Compact callus aggregate suspension cultures of *Rhodiola imbricata* are obtained on the MS medium supplemented with 3 mg/L NAA and 3 mg/L BAP [28]. *Rhodiola quadrifida* (Pall.) calluses are obtained from hairy roots in the MS medium with the addition of hormones: 3 mg/L 2,4-D and 0.5 mg/L BAP [31]. Calluses of *R. sachalinensis* Boriss. can be successfully cultivated on the MS medium supplemented with 3 mg/L BAP and 0.3 mg/L NAA [27]. The medium containing 1 mg/L 2,4-D, 2 mg/L NAA, 0.5 mg/L BAP, and 0.1 mg/L kinetin proved to be the best for the induction of the callus from *R. quadrifida* (the induction rate was 83.3%); the optimized combination of plant growth regulators for callus subculture is 1 mg/L 2,4-D, 0.1 mg/L BAP, and 0.5 mg/L kinetin [23]. Several other combinations of plant growth regulators have also been found to be effective for callus induction in species of the genus *Rhodiola*. One research group revealed that callus induction of *R. imbricata* is frequently achieved in juvenile leaves (100% frequency) and roots (87.50%) in the MS medium supplemented with 0.5 mg/L thidiazuron and 1 mg/L NAA [30]. In another study, to obtain a callus culture of *R. rosea*, leaves were placed on the surface of a fresh MS medium supplemented with 3 mg/L of N^6^-(2-isopentenyl) adenine and 0.3 mg/L IAA [29]. For callus subcultures and subsequent cell suspension cultures of *R. crenulata* L., full-strength MS containing 0.5 mg/L thidiazuron and 0.5 mg/L NAA turned out to be the best [26]. This paper optimized plant growth regulator proportioning, and maximum biomass and production of phenolic compounds were obtained in the medium containing BAP/NAA ratios of 0.33–1.00, provided that the concentration of plant growth regulators was 30 μM. Here, we found that optimal growth of a callus culture of *R. rosea* requires BAP > 0. Other reports suggest that the synergistic combinations of auxin and cytokinin can significantly alter the production of secondary metabolites depending on plant species [46]. When compared with the initial medium (control medium), total plant growth regulators content has not been changed. However, the selected complex of optimal factors, in general, contributed to a significant increase in the growth parameters of *R. rosea* calluses.

Here, the callus obtained under optimal culture conditions (NH_4_^+^/K^+^ 0.33, BAP/NAA 1.0, BAP + NAA 30 μM, and NO_3_^−^ 40 mM) was semi-friable yellowish green. The increase in the growth of fresh biomass on this medium was up to 703%, which is 2.7 times higher than the growth rates on the standard MS medium. Nonetheless, the level of TPC biosynthesis on the nutrient medium that gave the highest increase in callus biomass was 13% lower than that on the standard MS medium. The highest content of TPCs was observed in calluses grown on media with the NH_4_^+^/K^+^ ratio of 0.33 and BAP/NAA of 0.33 and a concentration of plant growth regulators of 30 μM. Data on the profile and concentrations of phenolic compounds in in vitro cultures vary and sometimes are contradictory because such results are influenced by various factors and stages of plant development [47]. Using RSM, it has been reported that higher KH_2_PO_4_ depletion and 75 μM m^−2^s^−1^ light intensity favored the biosynthesis of anthocyanins and the other phenolic compounds and resulted in elevated antioxidant capacity in grape (Bogazkere Cv.) callus culture [48]. Through Plackett–Burman’s design and RSM, optimal proportions of plant growth regulators for a cell suspension culture of *Siraitia grosvenorii* were obtained. With the optimized plant growth regulators, the obtained cell biomass and polyphenols content were 32.18% and 13.86%, respectively, more than plant growth regulators proportioning before optimization [49]. Using HPLC, we determined that the profile and levels of phenolic compounds were similar between the above-ground part of intact plants and the callus culture. TPC concentration varied among callus cultures from 14.9 to 71.6 mg/g, the number of phenolic compounds from 10 to 20; in the above-ground part, these values were 73.1 mg/g and 21 phenolic compounds, respectively. Several new phenolic compounds were identified in the callus cultures: compounds No. 9, 11, 19, 26, and 27 (see Table S1).

Jasmonic acid is thought to be involved in the signal transduction pathway that induces the production of defense compounds in plants, such as alkaloids, terpenoids, and polyphenols [50]. MJ is an effective elicitor that participates in plant defense response pathways and triggers plant metabolite biosynthesis. Accordingly, MJ has been used for inducing metabolite production in plant cell cultures [33]. In the present study, the medium was supplemented with different concentrations of MJ in 45-day-old cultures, and phenolic-compound accumulation was determined after three days of cultivation. The treatment of callus cultures with an elicitor led to qualitative and quantitative changes in the profile of phenolic compounds. In this case, MJ concentration was of paramount importance. The use of 100 μM MJ was optimal and led to an increase in the TPC content up to 47.9 mg/g. We showed that when callus cultures are exposed to an elicitor, phenolic compounds emerge that are absent in the sample without treatment; their retention times are 13.6, 30.0, 33.3, and 46.0 min. The effectiveness of MJ as an elicitor has been demonstrated for many in vitro cultures, including those of *Rhodiola* species [28,51,52,53,54,55,56,57]. For example, for *Rhodiola* it is reported that the levels of bioactive compounds increase with MJ supplementation in a dose-dependent manner. The highest salidroside content (4.75 mg/g dry weight) is obtained during treatment with MJ at 125 μM [28].

According to our HPLC analyses of rhizomes, the levels of rosarin, rosavin, rosin, and cinnamyl alcohol are 16.33, 41.73, 25.10, and 42.74 mg/g, respectively, while in the roots, their respective levels are 7.41, 11.73, 11.25 and 32.10 mg/g. In the aerial parts (leaves and stems), seedlings, and callus cultures, no detectable rosavins were found, in line with a report of Peschel et al. [58], wherein the aerial parts of wild *R. rosea*, no rosavins were identified, while the content of salidroside was below the detection limit. Although salidroside (0.53%) was found in the leaves of *R. rosea* from Rila Mountain, Bulgaria [24] and in the leaves and stems of *R. rosea* cultivated in Poland (salidroside 0.12% and total rosavins 0.3%), the aerial parts of the plant grow anew every year and therefore their content is consistent each time [59]. Rattan et al. [30] found that rosavin and rosarin are present at the highest concentration in root-derived compact green calluses (0.15 mg/g dry weight) and root-derived friable green calluses (0.07 mg/g dry weight). Kurkin et al. [60] noted that in a suspension culture of *R. rosea*, the main phenolic compound is a phenylpropanoid called triandrin, while in a callus culture, the process of biosynthesis went further and, together with triandrin, the major phenolic compounds were dimeric phenylpropanoids: lignans; in other words, “ageing” of the biomass took place. Those authors emphasized the finding that neither salidroside nor phenylpropanoids—which are characteristic for the rhizomes of roseroot stonecrop (rosin, rosavin, and rosarin)—were found in the samples of biomass. In the in vitro cultures, a significant enhancement in the production of rosin and its derivatives was observed when the cultures were fed with the precursor: cinnamyl alcohol [5]. In *R. rosea* compact callus aggregate cultures, the observed rosin and rosarin content was even higher than that in field-cultivated plants, while the rosavin level was five times lower [2]. Our next research project on the callus culture of *R. rosea* will be aimed at finding an optimal concentration and duration of cinnamyl alcohol treatment to promote the biosynthesis of phenylpropanoids.

## 4. Materials and Methods

### 4.1. Plant Material

*R. rosea* seeds were collected from its natural habitat on the southern slopes of the Iolgo ridge, Karakol lakes, Altai Republic (Russia). Altitude was 1800–2000 m a.s.l. *R. rosea* samples growing in this area belong to the ecotype of moderately humid habitats with moderate soil moisture (44–50%). This ecotype was previously chosen by us as optimal for the introduction experiment in the conditions of the forest-steppe zone of Western Siberia (Russia). Our earlier studies confirm the high biological and biosynthetic potential of these samples [61]. In vitro cultivation and ex vitro acclimatization of *R. rosea* plants were performed in the Laboratory of Biotechnology, CSBG SB RAS (Novosibirsk, Russia) according to the previously developed method [62].

### 4.2. Establishment of Callus Cultures

A callus line was developed from cotyledon explants of in vitro-grown seedlings of *R. rosea*. Seeds were germinated in the MS [63] basal medium without plant growth regulators but supplemented with 30 g/L sucrose and 6 g/L agar. For callus induction, cotyledon explants were excised from 21-day-old seedlings and placed onto the MS medium supplemented with 15 μM BAP (Sigma-Aldrich, St Louis, MO, USA), 15 μM NAA (Sigma-Aldrich), 30 g/L sucrose, and 6 g/L agar. The conditions of explant cultivation were as follows: photoperiod, 16/8-h light/dark cycle; illumination intensity, 2–3 klx; and temperature, 24 ± 1 °C. Callus cultures were maintained on the MS medium containing 15 μM BAP and 15 μM NAA via regular subculturing with a 45-day interval.

For the establishment of callus cultures at different media compositions, an actively growing 6-month-old callus was used as a source. The experiment lasted 45 days. Fresh and dry weights were measured by taking the average of three plates for each treatment type. Percent increase in fresh weight was calculated from the initial weight of the subcultured callus.

### 4.3. Optimal (Combined) Design

The experiment had a mixture–concentration design and included four mixture components (K^+^, NH_4_^+^, BAP, and NAA) and two numerical factors (NO_3_^−^ and BAP + NAA concentrations). As NH_4_^+^/K^+^ and BAP/NAA were regarded as components of mixture1 and mixture2, the range for each component was expressed as a proportion; all component proportions in each mixture add up to 1.0. NO_3_^−^ concentration ranged from 20 to 60 mM, K^+^ proportion from 0.5 to 1.0, and NH_4_^+^ proportion ranged from 0 to 0.5. BAP + NAA concentration ranged from 5 to 30 μM, NAA proportion from 0.5 to 1.0, and BAP proportion from 0 to 0.5. The concentration of K^+^ plus NH_4_^+^ matched the NO_3_^−^ concentration to maintain charge neutrality. Design points were selected via D-optimal criteria to satisfy a quadratic polynomial for the mixtures (NH_4_^+^/K^+^ and BAP/NAA) and the numerical factors (NO_3_^−^ and BAP + NAA) in various combinations in the mixture. The experiment included a data point for the MS basal medium.

All solution recipes were derived using the linear programming approach described by Niedz and Evens [64]. The concentrations of salts/acids/bases needed for each data point in the design space were calculated in the ARS-Media (Version 1.0) software (ion solution calculation program), which is available as a free download via http://www.ars.usda.gov/services/software/download.htm?softwareid=148 (accessed on 10 September 2020). For each treatment, all ions present and their concentrations were entered into ARS-Media. Ions other than those being varied were fixed at their normal levels present in the MS medium.

### 4.4. Effect of MJ Treatment

Callus cultures with the highest biomass growth were used for elicitation. Subsequently, a 45-day-old callus was transferred to a 100 mL Erlenmeyer flask containing 20 mL of a modified MS medium (NH_4_^+^/K^+^ 0.33, NO_3_^−^ 40 mM) supplemented with 15 μM NAA, 15 μM BAP, and different concentrations of MJ (100 or 200 μM). The modified MS medium without MJ served as a control. The culture flasks were placed on a rotary shaker (90 rpm, Elmi, S-3-02L, Latvia) at 24 ± 2 °C under a 16 h/8 h photoperiod. The accumulation of biomass and the production of phenolic compounds were implemented after 3 days of cultivation.

### 4.5. Biochemical Analysis

#### 4.5.1. Extraction

The dry raw material (callus cultures, in vitro seedlings, rhizomes, roots, and above-ground parts) was crushed to a particle size of 1 mm, mixed, and average samples were collected. Biochemical analysis was performed only on well-growing callus cultures with an increase in wet biomass of more than 300% and for callus grown on the standard MS medium. Double extraction was performed to isolate phenolic compounds. A 0.5 g sample of the crushed material was extracted with 30 mL of aqueous 50% ethanol for 8 h, and then with 20 mL of 70% ethanol for 50 min in a water bath. Each filter cake was washed with 5 mL of 70% ethanol. The combined extract was concentrated to 20 mL. To remove impurities, 1 mL of the extract was diluted with double-distilled water to 5 mL and passed through a Diapak C16 (ZAO BioKhimMak, Moscow, Russia) concentrating cartridge.

#### 4.5.2. HPLC Analysis

The profile of phenolic compounds in the samples was investigated by HPLC using an Agilent 1200 liquid chromatograph equipped with a diode array detector, Zorbax SB–C18 column (4.6 × 150 mm, with sorbent grain size 5 μm; Agilent Technologies, Santa Clara, CA, USA). In the mobile phase, the methanol content in the aqueous solution of phosphoric acid (0.1%) was varied from 50% to 52% within 56 min. The flow rate of the eluent was 1 mL/min; the column temperature was 26 °C and the volume of the injected sample was 10 μL. Detection was carried out at λ = 220, 250, 270, 290, 360, and 370 nm. Concentrations of substances were calculated by detection at 255 nm. Methyl alcohol (extra pure grade), orthophosphoric acid (extra pure grade), and double-distilled water were utilized to prepare mobile phases. Standards from Aobious (USA) and Sigma-Aldrich (Germany) were used for identification. Standard solutions were prepared at a concentration of 10 μg/mL in ethyl alcohol. The retention time of the peaks of compounds in the chromatograms of the analyzed samples and their UV spectra were compared with those of the standard samples. Quantitative analysis of individual phenolic compounds in plant samples was performed by the external standard method (% of the weight of air-dry raw materials).

To assess biosynthetic potential of the callus cultures, two controls were used in the study. Control 1 was 21-day-old in vitro seedlings of *R. rosea*. Control 2 consisted of cultivated 5-year-old *R. rosea* plants propagated in an in vitro culture [36]. The initial material for the control samples was seeds from the same population and the same year of collection as the experimental samples.

### 4.6. Histochemical Analysis

Callus cultures of *R. rosea* were fixed in a mixture of formalin, acetic acid, and 70% ethanol (7:7:100 *v*/*v*/*v*) for 4 days and then stored in 70% (*v*/*v*) ethanol. For fluorescent microscopy, fresh calluses were used.

For histochemical characterization, the calluses of *R. rosea* fixed as described above were sectioned (25−35 μm) on a MICROM HM 430 microtome (Thermo Fisher Scientific, Munich, Germany) with fast freezing unit KS 34 S (Thermo Fisher Scientific, Munich, Germany). For light microscopy, sections were analyzed by means of a Carl Zeiss Axioscope A1 microscope equipped with digital camera Axiocam 506 color, and the ZEN 2012 software (blue edition) (Carl Zeiss Ltd., Herts, UK) and Carl Zeiss Primo Star iLED equipped with a filter system (470 nm), digital camera AxioCam MRc, and the AxioVision 4.8 software.

Cross-sections were investigated by the following histochemical tests: a ferric chloride reaction to detect phenolic compounds and a reaction with Lugol’s solution to detect starch. For fluorescence microscopy, fresh sections were treated using a 2% (*w*/*v*) solution of safranin for 2 min to detect starch, inducing yellow color at 470 nm, respectively. For autofluorescence examination, the sections were directly viewed under LED light at 470 nm.

### 4.7. Statistical Data Analysis

Design-Expert^®^ 13 was used for experimental design construction, model evaluation, and all analyses. The data expressed as mean and standard error (M ± SE) were subjected to ANOVA in the STATISTICA 6.0 software. The differences between means were tested for significance by the LSD test at *p* ≤  0.05. In addition, clustering was performed by PCA.

## 5. Conclusions

In summary, this is the first report describing a statistical optimization study of biomass accumulation and the production of phenolic compounds in callus cultures of *R. rosea* using DOE. Computer-generated optimal design is an excellent tool for reducing treatment numbers compared to traditional factorial designs. The overall effect of the resulting model was highly significant (*p* < 0.0001), indicating that studied factors (NH_4_^+^, K^+^, NO_3_^−^, BAP, and NAA) significantly affected the growth of callus cultures of *R. rosea*. The best callus growth (703%) and the highest production of TPCs (75.17 mg/g) were achieved at an NH_4_^+^/ K^+^ ratio of 0.33 and BAP/NAA of 0.33, provided that the concentration of plant growth regulators was 30 μM and that of NO_3_^−^ was ≤40 mM. The information presented in this study may be useful for future research related to the cell culture of *R. rosea* and other *Rhodiola* species.

## Figures and Tables

**Table 1:** ANOVA and summary statistics for the % fresh weight increase and dry weights of callus culture of *R. rosea*.

	Fresh Weight		Dry Weight	
	F-Value	*p*-Value	F-Value	*p*-Value
Model	107.14	<0.0001	35.83	<0.0001
Linear × Linear Mixture	274.28	<0.0001	80.74	<0.0001
NH_4_^+^ * K^+^ *BAP	140.46	<0.0001	31.82	<0.0001
NH_4_^+^ * K^+^ *NAA	22.00	<0.0001	13.40	0.0008
NH_4_^+^ *BAP*NAA	38.42	<0.0001	6.61	0.0142
NH_4_^+^ *BAP* NO_3_^−^	5.78	0.0218	1.01	0.3208
NH_4_^+^ *BAP*BAP + NAA	21.64	<0.0001	6.87	0.0126
NH_4_^+^ * K^+^ *BAP* NO_3_^−^	19.65	<0.0001	-	-
NH_4_^+^ * K^+^ *BAP* BAP + NAA	19.84	<0.0001	2.74	0.1058
NH_4_^+^ * K^+^ *NAA* NO_3_^−^	7.92	0.0081	1.70	0.2002
NH_4_^+^ * K^+^ *NAA* BAP + NAA	15.35	0.0004	6.63	0.0141
NH_4_^+^ *BAP*NAA* NO_3_^−^	76.36	<0.0001	17.88	0.0001
NH_4_^+^ *BAP*NAA* BAP + NAA	68.45	<0.0001	-	-
NH_4_^+^ *BAP* NO_3_^−^* BAP + NAA	21.51	<0.0001	25.17	<0.0001
NH_4_^+^ * NAA* NO_3_^−^* BAP + NAA	12.34	0.0013	-	-
K^+^ *BAP* [NO_3_^−^]^2^	1.84	0.1842	-	-
NH_4_^+^ * K^+^ *BAP* NO_3_^−^* BAP + NAA	4.74	0.0364	7.54	0.0092
NH_4_^+^ *BAP*NAA* NO_3_^−^* BAP + NAA	23.44	<0.0001	1.83	0.1840
NH_4_^+^ * K^+^ *BAP*[ NO_3_^−^]^2^	61.34	<0.0001	16.24	0.0003
NH_4_^+^ * K^+^ *BAP*[ BAP + NAA]^2^	7.57	0.0095	3.79	0.0590
Lack of Fit	*p* = 0.8180		*p* = 0.6741	
R^2^	0.9851		0.9413	
Adjusted R^2^	0.9759		0.9150	
Predicted R^2^	0.9235		0.8162	
Adeq Precision	40.4608		23.0849	
Std. Dev.	25.21		0.0142	
Mean	178.00		0.1071	
C.V.%	14.16		13.26	
Model type	Reduced Quadratic × Quadratic × Quadratic model		Reduced Quadratic × Quadratic × Quadratic model	

Note: “-”: no hierarchical relationships.

**Table 2:** Characteristics and levels of the phenolic compounds detected by HPLC in the extracts from rhizomes and roots of *R. rosea*.

Compound	Spectral Characteristics: λ_max_, nm	Retention Time (t_R_), min	Content, mg/g of Air-Dried Material	
			Rhizomes	Roots
Gallic acid	272	1.8	26.15	21.20
Compound 2	216,280	2.9	3.20	2.30
Compound 3	228,296	6.5	0.51	0.23
Compound 4	218,274	8.5	0.41	-
Rosarin	253	10.7	16.33	7.41
Rosavin	253	12.5	41.73	11.73
Rosin	253	13.5	25.10	11.25
Cinnamyl alcohol	205,253	24.3	42.74	32.10
Rhodiosin	277,333,385	39.9	0.81	0.72
Rhodionin	277,333,385	40.5	2.11	1.74
TPC			159.09	88.68

“-”: not detected.

## Data Availability

Data available on request due to restriction.

## Supplementary Materials

The following supporting information can be downloaded at: https://www.mdpi.com/article/10.3390/plants11010124/s1, Figure S1: A chromatogram of a water-ethanol extract of the *R. rosea* callus culture that featured the highest TPC concentration, Figure S2: A chromatogram of a water-ethanol extract of the *R. rosea* callus culture cultivated under optimal nutrient conditions (A) with MJ 100 μM and (B) without MJ, Figure S3: A chromatogram of a water-ethanol extract of *R. rosea* rhizomes; Table S1: Characteristics and levels of the phenolic compounds detected by HPLC in the extracts from callus cultures and above-ground parts (herb) of *R. rosea* (mg/g).

## References

[1] Marchev A., Haas C., Schulz S. (2014). Sage in vitro cultures: A promising tool for the production of bioactive terpenes and phenolic substances. Biotechnol. Lett..

[2] Grech-Baran M., Sykłowska-Baranek K., Pietrosiuk A. (2015). Biotechnological approaches to enhance salidroside, rosin and its derivatives production in selected *Rhodiola* spp. in vitro cultures. Phytochem. Rev..

[3] Zheleznichenko T., Banaev E., Asbaganov S., Voronkova M., Kukushkina T., Filippova E., Mazurkova N., Shishkina L., Novikova T. (2018). *Nitraria schoberi* L. hairy root culture as a source of compounds with antiviral activity against influenza virus subtypes A(H5N1) and A(H3N2). 3 Biotech.

[4] Chiang H.M., Chen H.C., Wu C.S., Wu P.Y., Wen K.C. (2015). *Rhodiola* plants: Chemistry and biological activity. J. Food Drug Anal..

[5] Marchev A.S., Dinkova-Kostova A.T., György Z. (2016). *Rhodiola rosea* L.: From golden root to green cell factories. Phytochem. Rev..

[6] Khanna K., Mishra K.P., Ganju L., Singh S.B. (2017). Golden root: A wholesome treat of immunity. Biomed. Pharmacother.

[7] Brinckmann J.A., Cunningham A.B., Harter D.E.V. (2021). Running out of time to smell the roseroots: Reviewing threats and trade in wild *Rhodiola rosea* L.. J. Ethnopharmacol..

[8] György Z., Tolonen A., Pakonen M., Neubauer P., Hohtola A. (2004). Enhancement of the production of cinnamyl glycosides in CCA cultures of *Rhodiola rosea* through biotransformation of cinnamyl alcohol. Plant Sci..

[9] Ma L.Q., Gao D.Y., Wang Y.N., Wang H.H., Zhang J.X., Pang X.B., Hu T.S., Lu S.Y., Li G.F., Ye H.C. (2008). Effects of overexpression of endogenous phenylalanine ammonia-lyase (PALrs1) on accumulation of salidroside in *Rhodiola sachalinensis*. Plant Biol..

[10] György Z., Hohtola A., Jain S.M., Saxena P. (2009). Production of cinnamyl glycosides in compact callus aggregate cultures of *Rhodiola rosea* through biotransformation of cinnamyl alcohol. Protocols for In Vitro Cultures and Secondary Metabolite Analysis of Aromatic and Medicinal Plants. Methods in Molecular Biology.

[11] Martin J., Pomahacova B., Dusek J., Duskova J. (2010). In vitro culture establishment of *Schizandra chinensis* (Turz.) Baill. and *Rhodiola rosea* L., two adaptogenic compounds producing plants. J. Phytol..

[12] Furmanowa M., Hartwich M., Alfermann A.W. (1999). Rosavin as a product of glycosylation by *Rhodiola rosea* (roseroot) cell cultures. Plant Cell Tissue Organ Cult..

[13] Grech-Baran M., Sykłowska-Baranek K., Krajewska-Patan A. (2014). Biotransformation of cinnamyl alcohol to rosavins by non-transformed wild type and hairy root cultures of *Rhodiola kirilowii*. Biotechnol. Lett..

[14] Kapoor S., Raghuvanshi R., Bhardwaj P., Sood H., Saxena S., Chaurasia O.P. (2018). Influence of light quality on growth, secondary metabolites production and antioxidant activity in callus culture of *Rhodiola imbricata* Edgew. J. Photochem. Photobiol. B.

[15] Condra L.W. (2001). Reliability Improvement with Design of Experiments.

[16] Niedz R.P., Evens T.J. (2016). Design of experiments (DoE)—History, concepts, and relevance to in vitro culture. In Vitro Cell. Dev. Biol. Plant.

[17] Niedz R.P., Evens T.J. (2008). The effects of nitrogen and potassium nutrition on the growth of nonembryogenic and embryogenic tissue of sweet orange (*Citrus sinensis* (L.) Osbeck). BMC Plant Biol..

[18] Butenko R.G. (1964). The Culture of Isolated Tissues and the Physiology of Plant Morphogenesis.

[19] Ramage C.M., Williams R.R. (2002). Inorganic nitrogen requirements during shoot organogenesis in tobacco leaf discs. J. Exp. Bot..

[20] Cousson A., Van Tran Thanh K. (1993). Influence of ionic composition of the culture medium on de novo flower formation in tobacco thin cell layers. Can. J. Bot..

[21] Poothong S., Reed B.M. (2016). Optimizing shoot culture media for Rubus germplasm: The effects of NH_4_^+^, NO_3_^−^, and total nitrogen. In Vitro Cell. Dev. Biol. Plant.

[22] Niedz R.P., Evens T.J. (2007). Regulating plant tissue growth by mineral nutrition. In Vitro Cell. Dev. Biol. Plant.

[23] Sheng C.Z., Hu T.Q., Bi H., Yuan Y.J., Jiang Y. (2005). Effects of plant growth substances on induction and culture of callus from *Rhodiola quadrifida*. Zhongguo Zhong Yao Za Zhi.

[24] Tasheva K., Kosturkova G. (2010). Bulgarian golden root in vitro cultures for micropropagation and reintroduction. Cent. Eur. J. Biol..

[25] Ghiorghită G., Hârtan M., Maftei D. (2011). Some considerations regarding the in vitro culture of *Rhodiola rosea* L.. Rom. Biotechnol. Lett..

[26] Shi L., Wang C., Zhou X., Zhang Y., Liu Y., Ma C. (2013). Production of salidroside and tyrosol in cell suspension cultures of *Rhodiola crenulata*. Plant Cell Tiss. Organ. Cult..

[27] Li Y., Shao C.H., Park S.Y. (2014). Production of salidroside and polysaccharides in *Rhodiola sachalinensis* using airlift bioreactor systems. Acta Physiol. Plant.

[28] Kapoor S., Sharma A., Bhardwaj P. (2019). Enhanced production of phenolic compounds in compact allus Aggregate suspension cultures of *Rhodiola imbricata* Edgew. Appl. Biochem. Biotechnol..

[29] Mirmazloum I., Kiss A., Ladányi M. (2019). Production of cinnamyl alcohol glycosides by biotransformation in roseroot callus cells. Plant Cell Tiss. Organ Cult..

[30] Rattan S., Rattan S., Sood A., Kumar P., Kumar A., Kumar D., Kumar D., Warghat A.R., Warghat A.R. (2020). Phenylethanoids, phenylpropanoids, and phenolic acids quantification vis-à-vis gene expression profiling in leaf and root derived callus lines of *Rhodiola imbricata* (Edgew.). Ind. Crops Prod..

[31] Stepanova A., Malunova M., Salamaikina S., Selimov R., Solov’eva A. (2021). Establishment of *Rhodiola quadrifida* hairy roots and callus culture to produce bioactive compounds. Phyton-Int. J. Exp. Bot..

[32] Kolewe M.E., Gaurav V., Roberts S.C. (2008). Pharmaceutically active natural product synthesis and supply via plant cell culture technology. Mol. Pharm..

[33] Zhao J., Davis L.C., Verpoorte R. (2005). Elicitor signal transduction leading to production of plant secondary metabolites. Biotechnol. Adv..

[34] Gundlach H., Müller M.J., Kutchan T.M., Zenk M.H. (1992). Jasmonic acid is a signal transducer in elicitor-induced plant cell cultures. Proc. Natl. Acad. Sci. USA.

[35] Chu U.C., Adelberg J., Lowe K. (2019). Use of DoE methodology to optimize the regeneration of high-quality, single-copy transgenic *Zea mays* L. (maize) plants. In Vitro Cell. Dev. Biol. Plant.

[36] Erst A.A., Petruk A.A., Zibareva L.N., Erst A.S. (2021). Morphological, histochemical and biochemical features of cultivated *Rhodiola rosea* (Altai Mountains ecotype). Contemp. Probl. Ecol..

[37] Gómez-Montes E.O., Oliver-Salvador C., Durán-Figueroa N. (2015). Optimization of direct shoot regeneration using cotyledonary explants and true leaves from lettuce cv. Romaine (*Lactuca sativa* L.) by surface response methodology. Plant Growth Regul..

[38] Bagherieh-Najjar M.B., Nezamdoost T. (2016). Optimization of shikonin production in *Onosma dichroantha* callus using response surface methodology. Plant Cell Tiss. Organ Cult..

[39] Yu K.W., Hahn E.J., Paek K.Y. (2001). Effects of NH_4_^+^: NO_3_^−^ ratio and ionic strength on adventitious root growth and ginsenoside production in bioreactor culture of *Panax ginseng* C.A. Meyer. Acta Hortic..

[40] Chen Y.Q., Yi F., Cai M. (2003). Effects of amino acids, nitrate, and ammonium on the growth and taxol production in cell cultures of *Taxus yunnanensis*. Plant Growth Regul..

[41] Prakash G., Srivastava A.K. (2005). Statistical media optimization for cell growth and azadirachtin production in *Azadirachta indica* (A. Juss) suspension cultures. Process Biochem..

[42] Irshad M., Debnath B., Mitra S. (2018). Accumulation of anthocyanin in callus cultures of red-pod okra (*Abelmoschus esculentus* (L.) Hongjiao) in response to light and nitrogen levels. Plant Cell Tiss. Organ Cult..

[43] Oleszkiewicz T., Kruczek M., Baranski R. (2021). Repression of carotenoid accumulation by nitrogen and NH_4_^+^ supply in carrot callus cells in vitro. Plants.

[44] Martinez M.E., Jorquera L., Poirrier P., Díaz K., Chamy R. (2021). Effect of the carbon source and plant growth regulators (PGRs) in the induction and maintenance of an in vitro callus culture of *Taraxacum officinale* (L) weber Ex F.H. Wigg. Agronomy.

[45] Small C.C., Degenhardt D. (2018). Plant growth regulators for enhancing revegetation success in reclamation: A review. Ecol. Eng..

[46] Goyal S., Ramawat K.G. (2008). Synergistic effect of morphactin on cytokinin-induced production of isoflavonoids in cell cultures of *Pueraria tuberosa* (Roxb. ex. Willd.) DC. Plant Growth Regul..

[47] Muraseva D.S., Kostikova V.A. (2021). In vitro propagation of *Spiraea betulifolia* subsp. *aemiliana* (Rosaceae) and comparative analysis of phenolic compounds of microclones and intact plants. Plant Cell Tiss. Organ Cult..

[48] Karaaslan M., Ozden M., Vardin H., Yilmaz F.M. (2013). Optimisation of phenolic compound biosynthesis in grape (Bogazkere Cv.) callus culture. Afr. J. Biotechnol..

[49] Liu Z., Zhu X., Mohsin A., Yin Z., Zhuang Y., Zhou B., Du L., Yin X., Liu N., Wang Z. (2022). Embryogenic callus induction, cell suspension culture, and spectrum-effect relationship between antioxidant activity and polyphenols composition of Siraitia grosvenorii cultured cells. Ind. Crops Prod..

[50] Mizukami H., Tabira Y., Ellis B.E. (1993). Methyl jasmonate-induced rosmarinic acid biosynthesis in *Lithospermum erythrorhizon* cell suspension cultures. Plant Cell Rep..

[51] Shabania L., Ehsanpoura A.A., Asgharib G., Emamib J. (2009). Glycyrrhizin production by in vitro cultured *Glycyrrhiza glabra* elicited by methyl jasmonate and salicylic acid. Russ. J. Plant Physiol..

[52] Martin K.P., Sabovljevic A., Madassery J. (2011). High-frequency transgenic plant regeneration and plumbagin production through methyl jasmonate elicitation from hairy roots of *Plumbago indica* L.. J. Crop. Sci. Biotech..

[53] Ram M., Prasad K.V., Singh S.K., Hada B.S., Kumar S. (2013). Influence of salicylic acid and methyl jasmonate elicitation on anthocyanin production in callus cultures of *Rosa hybrida* L.. Plant Cell Tiss. Organ Cult..

[54] Chodisetti B., Rao K., Gandi S., Giri A. (2015). Gymnemic acid enhancement in the suspension cultures of *Gymnema sylvestre* by using the signaling molecules–methyl jasmonate and salicylic acid. In Vitro Cell. Dev. Biol. Plant.

[55] Erst A.A., Zibareva L.N., Filonenko E.S., Zheleznichenko T.V. (2019). Influence methyl jasmonate on production of ecdysteroids from hairy roots of *Silene linicola* C.C.Gmelin. Russ. J. Bioorg. Chem..

[56] Ho T.T., Murthy H.N., Park S.Y. (2020). Methyl jasmonate induced oxidative stress and accumulation of secondary metabolites in plant cell and organ cultures. Int. J. Mol. Sci..

[57] Erst A.A., Zibareva L.N., Filonenko E.S. (2021). Variation in phytoecdysteroid accumulation in hairy roots of *Silene linicola* over extended time periods. J. Plant Biochem. Biotechnol..

[58] Peschel W., Kump A., Horváth A., Csupor D. (2016). Age and harvest season affect the phenylpropenoid content in cultivated European *Rhodiola rosea* L.. Ind. Crops Prod..

[59] Kołodziej B., Sugier D. (2013). Influence of plant age on the chemical composition of roseroot (*Rhodiola rosea* L.). Acta Sci. Pol. Hortorum. Cultus.

[60] Kurkin V.A., Zapesochnaya G.G., Dubichev A.G. (1991). Phenylpropanoids of a callus culture of *Rhodiola rosea*. Chem. Nat. Compd..

[61] Erst A.A., Yakubov V.V. (2019). Regenerative in vitro capacity of rare species *Rhodiola rosea* L. from various habitats. Contemp. Probl. Ecol..

[62] Erst A., Erst A., Shmakov A. (2018). In vitro propagation of rare species *Rhodiola rosea* from Altai Mountains. Turczaninowia.

[63] Murashige T., Skoog F. (1962). A revised medium for rapid growth and bioassays with tobacco tissue cultures. Physiol. Plant.

[64] Niedz R.P., Evens T.J. (2006). A solution to the problem of ion confounding in experimental biology. Nat. Methods.

