# Supplementary material for: Optimization of Biomass Accumulation and Production of Phenolic Compounds in Callus Cultures of Rhodiola rosea L. Using Design of Experiments
Source: Plants (Basel). 2022 Jan 2;11(1):124. doi: 10.3390/plants11010124 (PMC8747766; doi:10.3390/plants11010124)
Supplement: Supplementary file 1 [file plants-11-00124-s001.zip › plants-1512191-supplementary.pdf]

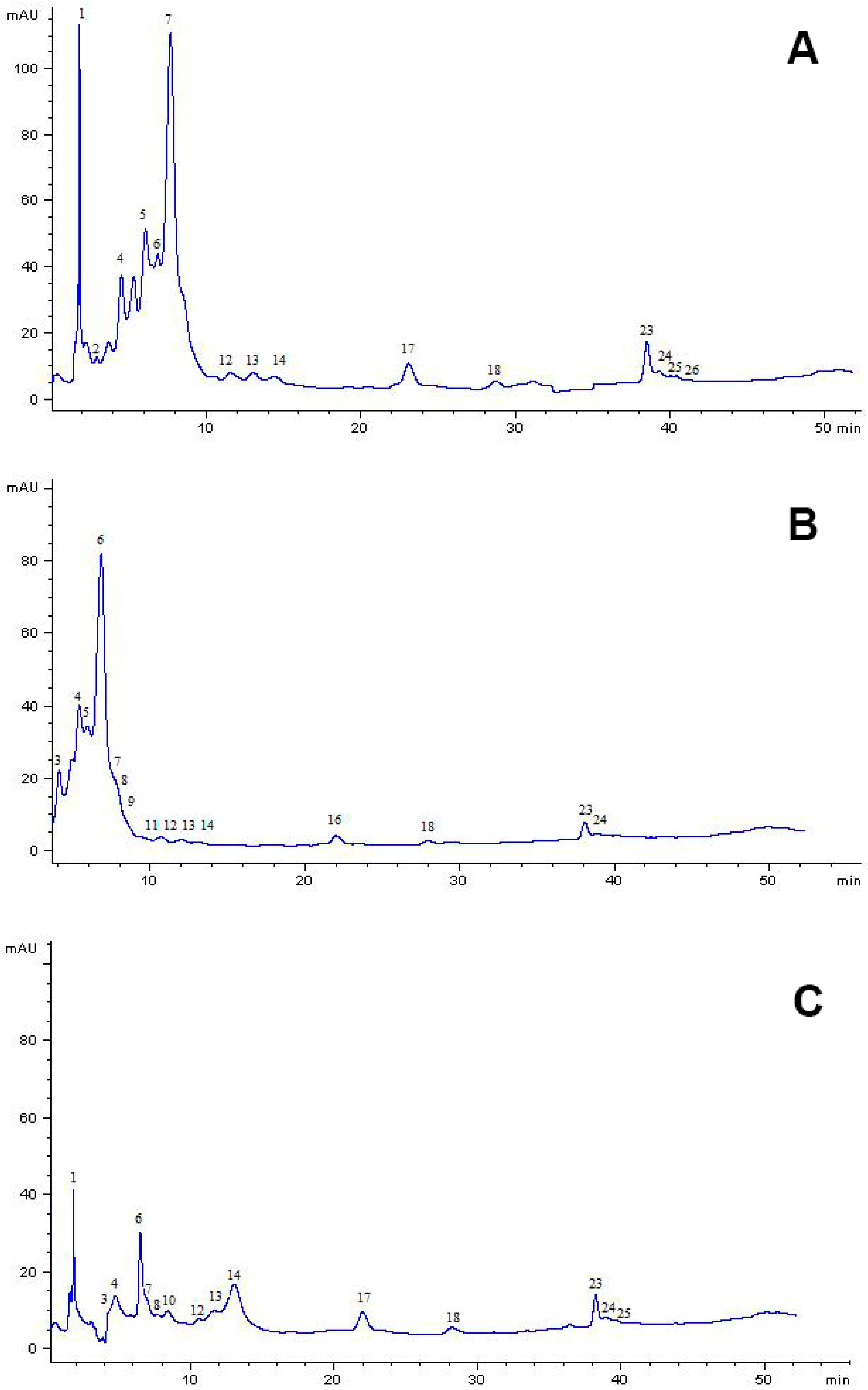

Figure S1. A chromatogram of a water-ethanol extract of the *R. rosea* callus culture that featured the highest TPC concentration. (**A**) NH_4^+/K^+ 0.33, NO_3^- 20 mM, BAP/NAA 0.33, BAP+NAA 30 μM. (**B**) NH_4^+/K^+ 0.33, NO_3^- 60 mM, BAP/NAA 0.33, BAP+NAA 30 μM. (**C**) NH_4^+/K^+ 0.33, NO_3^- 40 mM, BAP/NAA 0.33, BAP+NAA 30 μM. Note: The X-axis is retention time (t), min; Y-axis: optical density, arbitrary units (mAU). The numbers in the chromatogram indicate ID numbers of phenolic compounds (see Table S1).

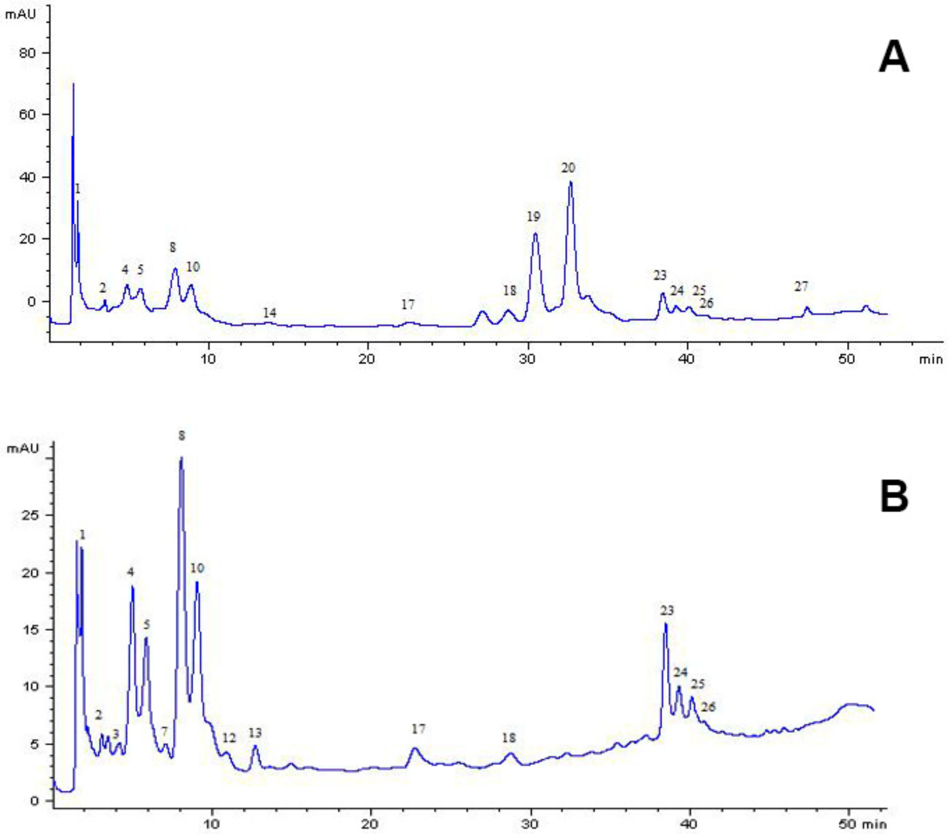

Figure S2. A chromatogram of a water-ethanol extract of the *R. rosea* callus culture cultivated under optimal nutrient conditions (**A**) with MJ 100 µM and (**B**) without MJ. Note: The X-axis is retention time (t), min; Y-axis: optical density, arbitrary units (mAU). The numbers in the chromatogram indicate ID numbers of phenolic compounds (see Table S1).

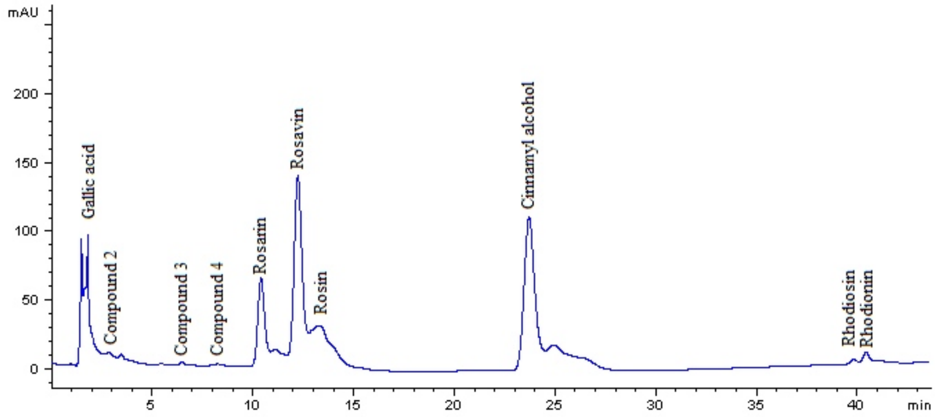

Figure S3. A chromatogram of a water-ethanol extract of *R. rosea* rhizomes. The X-axis is retention time (t), min; the Y-axis is optical density, arbitrary units (mAU).

Table S1. Characteristics and levels of the phenolic compounds detected by HPLC in the extracts from callus cultures and above-ground parts (herb) of *R. rosea* (mg/g)

**Compound ID**	**Spectral characteristics: λ_{max}, nm**	**Retention time (t_R), min**	**Treatment group ID (boldfaced header), showing concentrations below, mg/g of air-dried material**																
			**1**	**2**	**3**	**4**	**5**	**6**	**7**	**8**	**9**	**10**	**11**	**12**	**13**	**14**	**15**	**16**	**Herb**
1	272	1.8	1.39	5.21	3.53	3.45	6.35	3.07	4.52	2.09	3.17	3.20	2.25	3.48	3.37	3.11	4.24	–	2.53
2	216, 280	2.9	0.51	2.13	0.61	0.70	–	1.14	3.58	1.08	–	2.28	0.84	2.27	2.15	1.19	1.15	–	0.94
3	242, 340	4.1	0.52	–	5.14	0.84	0.52	1.43	5.03	0.71	–	6.50	0.60	1.34	2.15	–	–	–	0.91
4	242, 340	4.6	1.51	8.41	7.28	7.13	4.02	5.01	6.17	3.44	5.27	4.17	1.48	1.45	3.35	4.23	2,10	0.32	1.30
5	242, 332	6.0	1.05	18.10	–	3.12	–	5.23	7.13	3.07	1.28	–	–	3.02	2.17	3.01	2.43	–	0.41
6	228, 296	6.5	0.15	7.15	23.02	–	8.38	–	28.11	0.62	1.09	14.07	0.92	4.29	5.23	–	–	–	5.92
7	244, 340	7.1	0.23	18.23	4.16	–	2.25	1.02	5.07	–	–	–	0.81	2.44	4.16	–	–	0.31	5.01
8	242, 350	7.8	1.08	–	–	–	0.63	9.28	2.35	4.03	4.11	–	–	3.22	–	5.31	5.22	0.20	2.06
9	270, 352	8.3	–	–	–	–	–	–	4.20	–	–	–	–	–	–	–	–	–	–
10	218, 274	8.5	1.64	–	–	–	2.59	6.47	–	3.32	6.27	–	1.53	3.47	6.21	4.17	3.01	0.31	10.53
11	270	9.2	–	–	–	–	–	–	2.10	–	–	–	–	–	–	–	–	–	–
12	216, 280	10.8	0.40	2.14	0.40	–	1.07	0.82	0.41	–	2.51	0.10	1.42	1.63	1.27	–	–	–	1.04
13	216, 280	12.0	0.41	2.33	0.41	–	3.17	0.65	0.32	–	3.20	0.11	2.09	5.39	3.41	–	–	–	9.50
14	216, 280	13.6	0.20	1.28	0.31	–	13.08	–	0.20	0.50	16.09	0.10	2.15	8.57	10.24	0.31	–	–	23.01
15	220, 288	20.0	0.22	–	–	–	–	–	–	–	–	–	1.43	0.42	1.45	–	–	–	0.72
16	220, 288	31.3	–	–	–	–	–	–	1.21	–	–	–	–	1.18	0.52	–	–	–	0.70
17	256, 376	22.2	1.09	3.53	1.46	1.03	4.35	0.83	–	0.51	2.01	0.31	5.22	–	0.51	0.44	–	–	1.32
18	255	28.2	2.59	1.10	0.77	0.72	2.17	0.61	0.41	0.50	1.02	0.41	0.63	0.40	0.65	2.27	0.71	–	1.30
19	254	30.0	–	–	–	–	–	–	–	–	–	–	–	–	–	8.41	5.27	–	–
20	252	33.3	–	–	–	–	–	–	–	–	–	–	–	–	0.32	12.10	6.00	–	0.42
21	270, 325	35.4	–	–	–	–	–	–	–	–	–	–	–	–	–	–	–	1.38	–
22	216, 276	36.3	–	–	–	–	–	–	–	–	1.07	–	0.20	–	–	–	–	–	0.33
23	256, 379	38.1	2.14	3.40	1.03	3.23	4.53	3.51	0.91	2.50	2.44	0.31	5.07	0.44	0.81	2.45	1.37	2.07	2.64
24	256, 379	38.9	2.21	1.53	0.43	1.26	1.68	2.04	0.55	0.86	–	–	2.47	2.16	0.80	1.17	1.09	0.42	1.61
25	256, 379	39.7	0.43	0.33	0.40	0.91	1.22	1.22	–	0.50	0.52	–	0.74	1.15	0.54	1.09	1.13	1.32	1.0
26	220, 360	40.4	–	0.30	–	–	–	0.52	–	–	0.50	–	–	0.41	0.42	0.51	–	–	–
27	255, 270	46.0	–	–	–	–	–	–	–	–	–	–	–	–	–	0.72	0.51	1.17	–
**TPC**			**17.77**	**75.17**	**48.95**	**22.39**	**56.01**	**42.85**	**72.27**	**23.73**	**50.55**	**31.56**	**29.85**	**46.73**	**49.73**	**50.49**	**34.23**	**7.50**	**73.20**

1: Gallic acid, "–": not detected. *Legend* (see Figure 3).
